# A comparison of empiric therapy with cefazolin versus ceftriaxone for patients with complicated urinary tract infections in a tertiary care veterans affairs medical center

**DOI:** 10.1186/s12879-025-10494-5

**Published:** 2025-03-03

**Authors:** Paola Carcamo, Elizabeth Walter, Christopher R. Frei, Linda Yang, Jose Cadena, Teri Hopkins

**Affiliations:** 1 South Texas Veterans Health Care System, San Antonio, TX USA; 2 Pharmacotherapy Education & Research Center, University of Texas Health at San Antonio, San Antonio, TX USA; 3 University of Texas Austin College of Pharmacy, Austin, TX USA; 4 Division of Infectious Diseases, Department of Medicine, University of Texas Long School of Medicine, San Antonio, TX USA

**Keywords:** Complicated urinary tract infections, Cefazolin, Ceftriaxone, Inpatient, Antimicrobial stewardship

## Abstract

**Background:**

Data are limited regarding use of narrow-spectrum agents for the treatment of complicated urinary tract infections (cUTIs). We sought to evaluate cefazolin compared with ceftriaxone for the empiric treatment of patients with cUTIs in an inpatient setting.

**Methods:**

We conducted a retrospective, single-center, cohort study involving patients with cUTI treated with cefazolin or ceftriaxone at a Veterans Affairs (VA) medical center between November 1, 2019 and September 30, 2022. The primary outcome was 30-day clinical success, defined as resolution of signs and symptoms of infection without re-initiation of antibiotics during hospitalization or relapse within 30 days after cUTI diagnosis. Secondary outcomes included hospital length of stay and *Clostridioides difficile* infection (CDI) within 30 days of the end of antibiotic therapy.

**Results:**

We identified 113 patients with cUTI treated with cefazolin (*n* = 52) or ceftriaxone (*n* = 61) meeting study criteria. The study arms had similar demographics, although patients treated with ceftriaxone more frequently had subjective fever on admission or nephrolithiasis while cefazolin-treated patients had more altered mental status as the only UTI symptom reported, urinary catheter, and had a single dose of another antibiotic prior to starting the study medication. Clinical success was achieved in 47/52 (90%) and 53/61 (87%) in the cefazolin and ceftriaxone groups, respectively (*P* = 0.56). Additionally, there were no statistically significant differences in length of stay and development of CDI.

**Conclusions:**

In this retrospective cohort study of patients with cUTI at a VA medical center, empiric therapy with cefazolin appears to be a safe and effective treatment option.

**Supplementary Information:**

The online version contains supplementary material available at 10.1186/s12879-025-10494-5.

## Introduction

The Infectious Diseases Society of America (IDSA) has developed clinical practice guidelines for catheter-associated urinary tract infections as well as uncomplicated cystitis and pyelonephritis in women, which primarily addresses young women in the outpatient setting [1, 2]. Currently, there are no published United States guidelines for the treatment of cUTIs in non-catheterized men. In clinical practice, third-generation cephalosporins (i.e., ceftriaxone) are commonly used as empiric therapy. However, third-generation cephalosporins have been associated with a high risk of developing *Clostridioides difficile* infection (CDI) and the emergence of colonization or infection with multidrug-resistant organisms, such as extended-spectrum beta-lactamase (ESBL)-producing organisms. In contrast, the use of more narrow-spectrum beta-lactams, such as cefazolin, has been associated with less collateral damage [3, 10, 11]. 

There are limited data on the clinical use of first-generation cephalosporins in the hospital setting for the treatment of patients with cUTIs. A retrospective cohort study conducted by Hobbs et al. found cefazolin to be non-inferior to ceftriaxone for a composite of symptomatic resolution of acute pyelonephritis in hospitalized patients (87% vs. 86%, 95% CI, -11.1–8.9; *P* = 0.83).^4^ Another small, single-center, single-blinded, prospective study conducted in Iran, found cefazolin to be non-inferior to ceftriaxone for clinical response of pyelonephritis (81% vs. 91%; *P* = 0.21).^5^ A more recent systematic review has provided evidence of the effectiveness of first-generation cephalosporins in the treatment of patients with cUTI [6]. Cefazolin was the first-generation cephalosporin of choice in 6 of 7 studies, although the dose, frequency, and route of administration varied between the studies [6]. The authors found no difference in several outcomes including clinical cure, length of hospital-stay, and reinfection when comparing first-generation cephalosporins with other antimicrobials [6]. The quality of evidence was low, however, due to a small sample size, risk of bias, and imprecision for several of the analyzed outcomes. The current literature provides promising evidence for the use of narrow-spectrum beta-lactams for the treatment of patients with cUTIs; however, data in male patients are still limited.

In 2021, our local institution’s susceptibilities for the most common urinary tract pathogens (i.e., *Escherichia coli*,* Klebsiella pneumoniae*,* Proteus mirabilis*) were similar for cefazolin and ceftriaxone (Supplementary Table 1). Additionally, cefazolin concentrates in the urine exhibiting a favorable pharmacokinetic profile for the treatment of UTI [7]. Based on these factors and as an antimicrobial stewardship initiative to reduce third-generation cephalosporin use, our local inpatient clinical pathway for the treatment of UTIs was updated in early 2021. The pathway recommends intravenous (IV) cefazolin at a dose of 1–2 g every 8 h as the first-line empiric treatment regimen for patients without history of cefazolin-resistant isolates in culture, with consideration for higher dosing of 2 g IV every 8 h if there is a clinical suspicion for bacteremia. Cefazolin dosing was adjusted based on the patient’s renal function (calculated as creatinine clearance) in accordance with a local dosing protocol. This study aims to evaluate cefazolin compared with ceftriaxone for the empiric treatment of cUTIs in an inpatient setting.

## Methods

### Study design

This single center, retrospective, cohort study evaluated patients admitted at the South Texas Veterans Health Care System between November 1, 2019 through September 30, 2022. The University of Texas Health at San Antonio Institutional Review Board (San Antonio, TX, USA) conducted a review through the Office of Clinical Research. The study was approved under non-regulated research, exempt from Institutional Review Board review, and classified as a quality improvement project. Clinical trial number: not applicable.

### Data collection

ICD-10-CM diagnostic codes for admissions related to cUTI or pyelonephritis were used to identify patients for inclusion during the study period. Review of the electronic medical record was used to confirm cUTI diagnosis based on symptoms or available imaging to validate application of ICD-10-CM codes and consideration for inclusion. Data were obtained from the Veterans Health Administration’s (VHA) Corporate Data Warehouse (CDW). Data on demographics, preexisting medical conditions, microbiological results, antibiotic treatments, and outcomes were collected by manual chart review.

### Definitions

Complicated urinary tract infections (UTIs) were defined as UTIs associated with obstruction, foreign bodies, or urologic abnormalities, male UTIs and acute pyelonephritis with at least one UTI symptom reported. UTI symptoms included dysuria, urine frequency, urgency, hematuria, suprapubic pain, flank pain, fever, malaise, and altered mental status. A pragmatic approach was chosen to include patients with altered mental status as the only UTI symptom if the decision was made to treat with antibiotics. Female patients were additionally required to have current bladder catheterization, instrumentation of the urinary tract, or functional or anatomical abnormality of the urinary tract to be included in the study. Pyelonephritis included the same criteria as cUTI, plus fever and flank pain/costovertebral angle tenderness. Imaging-confirmed pyelonephritis was defined by compatible images on CT or ultrasound.

Immunosuppression was defined as steroids at > 20 mg prednisone equivalent per day for ≥ 14 days before the diagnosis of UTI, chemotherapy within 3 months before the diagnosis of UTI, receipt of immuno-modulators, transplantation, or advanced HIV/AIDS (CD4 count < 200 cells/mm^3^ or CD4% <14%).

### Study population

Patients were included if they were 18 years of age or older, had an admission diagnosis of cUTI, and received empiric treatment with ≥ 24 h of IV cefazolin or ceftriaxone.

Patients were excluded for the following reasons: (1) ICU admission, (2) hospice or comfort measures only, (3) complete obstruction of urinary tract, perinephric or intrarenal abscess, prostatitis, (4) received > 1 dose of another antibiotic prior to study drug with allowance for one dose of any antibiotic including ceftriaxone in emergency department, (5) concomitant infections besides bacteremia associated with UTI, (6) pregnant, (7) hemodialysis, (8) neutropenia, (9) repeat encounters with readmissions ≤ 30 days, or (10) only altered mental status reported without a positive urine culture.

### Outcomes

The primary outcome was 30-day clinical success, defined as resolution of signs and symptoms of cUTI, without re-initiation of antibiotics during hospitalization or relapse, at 30 days after cUTI diagnosis (e.g., date of the index urine culture for that episode of infection). Relapse required treatment for re-presentation of cUTI with a repeat urine culture growing an isolate identical to the index urine culture – isolates could have different susceptibility profiles. If the index urine culture was not available or isolated (1) no growth, (2) normal flora, or (3) was polymicrobial with ≥3 species, then any isolate in repeat culture represented a relapse. Secondary outcomes included hospital length of stay (LOS) and CDI within 30 days of the end of antibiotic therapy.

### Statistical analysis

Descriptive statistics were used to describe baseline characteristics; counts and percentages were used for categorical data and median and interquartile range (IQR) were used for numerical data. Numerical variables were compared using the Student’s *t* test for normally distributed numerical data and the Mann Whitney U test for skewed numerical data. Categorical variables were compared using the chi-square test.

The sample size estimate was calculated a priori using previously published data [5]. Using a one-sided test, and assuming a control group event rate of 86%, a sample size of 56 patients was needed in each treatment group to achieve 80% power to detect a 15% difference between groups.

We conducted a multivariate logistic regression analysis for treatment group (independent variable) and the outcome of 30-day clinical success (dependent variable). Multivariable analysis was chosen given the clinically relevant predictors we believed a priori to be important potential covariates for clinical success; these included subjective fever, urinary catheter, nephrolithiasis, bacteremia, and receipt of another antibiotic. Adjusted odds ratios were estimated with 95% CIs.

## Results

**Fig. 1:**
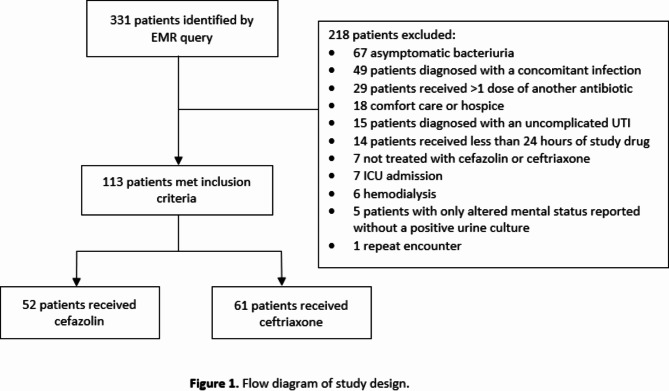
Flow diagram of study design

A total of 331 patients were identified and 218 were excluded, leaving 113 patients (52 in the cefazolin group and 61 in the ceftriaxone group) who met study criteria. The most common reasons for exclusion were patients with asymptomatic bacteriuria, concomitant non-urinary tract infections, and receipt of more than one dose of another antimicrobial prior to receiving the study drug (Fig. 1).

### Patient and infection-related characteristics

The median age was 72 years (IQR, 65–75) and 110 (97%) patients were male. Less than one-quarter of the cohort (12%) had immunocompromising conditions and 99 (88%) had underlying urologic abnormalities. More patients in the ceftriaxone group reported the following UTI symptoms on admission, 75% had urinary symptoms, 59% had subjective fever, and 56% had malaise/fatigue; for the cefazolin group, these percentages were 58%, 35%, and 31%, respectively. In contrast, the cefazolin group had more patients with altered mental status (37% vs. 18%, *P* = 0.03). Additionally, the cefazolin group had more urinary catheters compared to the ceftriaxone group (39% vs. 21%, *P* = 0.05). Significantly more patients in the cefazolin group also received a single dose of another antibiotic prior to starting the study medication (50% vs. 26%, *P*  =  0.01). Of those in the cefazolin group, 19 patients (37% of the total) received ceftriaxone. The remaining baseline characteristics were similar between the two groups (Table 1).

**Table 1:** Baseline characteristics

Characteristic	CFZ (*n* = 52)	CRO (*n* = 61)	*P* value
Age, years (IQR)	74 (64–75)	71 (66–75)	0.77
Male (%)	50 (96.2)	60 (98.4)	0.59
BMI, kg/m^2^ (IQR)	27 (23–33)	29 (24–38)	0.07
Diabetes mellitus (%)	30 (57.7)	30 (49.2)	0.37
Cirrhosis (%)	3 (5.8)	9 (14.8)	0.11
**Immunocompromised (%)**	5 (9.6)	9 (14.8)	0.41
Transplant	1 (1.9)	2 (3.3)	1.0
Oncology	4 (7.7)	7 (11.5)	0.50
HIV	0 (0)	0 (0)	1.0
Benign prostatic hyperplasia (%)	19 (36.5)	30 (49.2)	0.18
Spinal cord injury (%)	4 (7.7)	5 (8.2)	1.0
**Labs/vitals on admission (IQR)**			
Temperature, ^◦^F	98.3 (97.8–99.1)	98.4 (97.7–99.7)	0.87
WBC count, ×10^3^ cells/mL	10.6 (6.5–15.8)	11.7 (7.3–15.9)	0.56
Creatinine, mg/dL	1.1 (0.9–1.5)	1.3 (1.1–1.9)	0.08
Pyelonephritis (%)	7 (13.5)	7 (11.5)	0.75
CT/US-confirmed pyelonephritis (%)	6 (11.5)	6 (9.8)	0.77
Bacteremia (%)	5 (14.3)	6 (12.0)	0.75
**Symptoms of UTI on admission (%)**			
Urinary symptoms^a^	30 (57.7)	46 (75.4)	**0.05**
Subjective fever	18 (34.6)	36 (59.0)	**0.01**
Flank pain	10 (19.2)	8 (13.1)	0.38
Malaise/fatigue	16 (30.8)	34 (55.7)	**0.01**
Altered mental status	19 (36.5)	11 (18.0)	**0.03**
**Urinary analysis on admission (%)**			
Leukocyte esterase positive	47 (92.2)	59 (96.7)	0.41
Nitrite positive	20 (39.2)	18 (29.5)	0.28
Bacteria present	38 (74.5)	37 (60.7)	0.12
Pyuria (> 10 WBC/mm^3^)	44 (86.3)	61 (100)	**0.003**
**Urologic abnormalities (%)**			
Urinary catheter	20 (38.5)	13 (21.3)	**0.05**
Nephrolithiasis	5 (9.6)	13 (21.3)	0.08
Ureteral stent	0 (0)	4 (6.7)	0.12
Other urologic/functional abnormalities^b^	16 (30.8)	28 (45.9)	0.09
**Receipt of another antibiotic prior to study drug (%)**	26 (50.0)	16 (26.2)	**0.01**
Cefazolin	--	3 (4.9)	
Ceftriaxone	19 (36.5)	--	
Piperacillin/tazobactam	3 (5.8)	5 (8.2)	
Other^c^	4 (7.7)	8 (13.1)	
Antibiotics switched due to susceptibilities (%)	5 (9.6)	6 (9.8)	0.97
Duration of IV antibiotics, days (IQR)	2.9 (2.2–4.3)	3.0 (2.0-3.9)	0.74
Duration of therapy post-discharge, days (IQR)	4.5 (2.3-5.0)	5.0 (3.5-7.0)	0.13
Total duration of therapy, days (IQR)	7.6 (6.0-8.5)	7.9 (7.0-7.9)	0.1

Source: Supplementary Table 1

^a^Urinary symptoms: dysuria, frequency, urgency, hematuria, suprapubic pain

^b^Urologic/functional abnormalities include neurogenic bladder (*n* = 6 for cefazolin; *n* = 8 for ceftriaxone), urethral strictures (*n* = 1 for cefazolin; *n* = 2 for ceftriaxone), renal cysts (*n* = 1 for cefazolin; *n* = 5 for ceftriaxone), urologic cancers (prostate cancer [*n* = 6 for cefazolin; *n* = 5 for ceftriaxone], bladder cancer [*n* = 3 for ceftriaxone]), urethral erosion (*n* = 1 for cefazolin), renal transplant (*n* = 1 for cefazolin; *n* = 1 for ceftriaxone), other (*n* = 4 for ceftriaxone)

^c^Other antibiotics include amoxicillin/clavulanate, cefepime, fluoroquinolones, vancomycin

A total of 111/113 (98%) patients had urine cultures collected; two patients in the cefazolin group did not have a urine culture ordered. *E. coli* was the predominant pathogen isolated in both groups, accounting for 13 (26%) and 20 (33%) in the cefazolin and ceftriaxone groups, respectively. A total of 20 (18%) patients had urine cultures with no growth, of which 7/11 (64%) in the cefazolin group and 4/9 (44%) in the ceftriaxone group received antibiotics prior to urine culture collection. A total of 9 (8%) patients had urine cultures with normal flora, of which 4/5 (80%) cefazolin patients and 2/4 (50%) ceftriaxone patients received antibiotics prior to urine culture collection. Of the positive blood cultures, *E. coli* accounted for 2/5 (40%) and 4/6 (67%) in the cefazolin and ceftriaxone groups, respectively. One isolate in the cefazolin group and three in the ceftriaxone group were ESBL-producing organisms. Table 2 includes a complete description of the isolated organisms.

**Table 2:** Urine and blood culture organisms

	CFZ	CRO
**Urine culture** ^**a**^ **(%)**	*N* = 50	*N* = 61
*Escherichia coli*	13 (26.0)	20 (32.8)
*Proteus mirabilis*	2 (4.0)	4 (6.6)
*Klebsiella pneumoniae*	3 (6.0)	6 (9.8)
Mixed growth with < 2 species	3 (5.6)	6 (9.8)
Polymicrobial with ≥3 species^b^	6 (11.1)	6 (9.8)
No growth^c^	11 (22.0)	9 (14.8)
Normal flora^d^	5 (9.3)	4 (6.6)
*Staphylococcus epidermidis*	1 (1.9)	1 (1.6)
*Staphylococcus aureus*	2 (3.7)	--
*Enterococcus faecalis*	--	1 (1.6)
*Proteus vulgaris*	1 (1.9)	1 (1.6)
*Enterobacter cloacae complex*	--	1 (1.6)
*Aerococcus urinae*	--	1 (1.6)
*Pseudomonas aeruginosa*	1 (1.9)	--
*Candida spp.*	1 (1.9)	1 (1.6)
ESBL-producing organism	1 (1.9)	3 (4.9)
**Blood culture (%)**	***N*** = **5/35**	***N*** = **6/50**
*E. coli*	2 (40.0)	4 (66.7)
*P. mirabilis*	--	1 (16.7)
*K. pneumoniae*	--	1 (16.7)
*P. vulgaris*	1 (20.0)	--
*S. epidermidis*	1 (20.0)	--
Mixed growth	1 (20.0)	--

^a^Of note, 3/27 (11.1%) identified isolates with available antimicrobial susceptibility testing were resistant to cefazolin and 5/42 (11.9%) were resistant to ceftriaxone

^b^Polymicrobial with ≥3 species is considered probable contaminant by lab and organisms are not worked up

^c^7/11 (63.6%) cefazolin patients and 4/9 (44.4%) ceftriaxone patients received antibiotics prior to urine culture collection

^d^4/5 (80%) cefazolin patients and 2/4 (50%) ceftriaxone patients received antibiotics prior to urine culture collection

Antibiotic doses were as follows. In the cefazolin group, 36 (69%) patients received 1 gram IV every 8 h and 9 (17%) patients received 2 g IV every 8 h. Appropriate renal dose adjustments were made for patients in the cefazolin group due to reduced creatinine clearance; 6 (12%) patients received 1 gram IV every 12 h and 1 (2%) patient received 2 g IV every 12 h. In the ceftriaxone group, 49 (80%) patients received 1 gram IV every 24 h and 12 (20%) patients received 2 g IV every 24 h. The median durations of cefazolin (2.9, 2.2–4.3 days) and ceftriaxone (3.0, 2.0–3.9 days) were similar (*P* = 0.74).

Outpatient discharge orders were evaluated to determine whether there was a difference between the cefazolin and ceftriaxone groups. There were 46 (88%) patients in the cefazolin group and 55 (90%) patients that transitioned to oral therapy. Patients received a beta-lactam (36/52 vs. 40/61), a fluoroquinolone (5/52 vs. 10/61), sulfamethoxazole/trimethoprim (3/52 vs. 5/61), doxycycline (2/52 vs. 0/61) or no antibiotics (6/52 vs. 6/61) at discharge in the cefazolin and ceftriaxone groups, respectively. The discharge antibiotic regimens were similar between the two groups with beta-lactams being the primary agents used (Supplementary Table 2). The ceftriaxone group used more fluoroquinolones compared to cefazolin, these percentages were 16% and 10%, respectively. The median durations of oral antibiotic therapy for cefazolin (4.5, IQR, 2.3–5.0 days) and ceftriaxone (5.0, IQR, 3.5–7.0 days) were similar (*P* = 0.13).

### Clinical outcomes

Clinical success was achieved in 47/52 (90%) and 53/61 (87%) in the cefazolin and ceftriaxone groups, respectively (*P* = 0.56) (Table 3). Multivariate analysis identified urinary catheters as an independent, negative predictor of clinical success, with an odds ratio of 0.24 (95% CI, 0.06–0.89). Cefazolin use was included in the multivariate logistic regression analysis to assess whether the use of cefazolin would predict achievement of the primary outcome. Cefazolin remained a non-significant predictor of clinical success (Table 4 and Supplementary Fig. 1).

**Table 3:** Clinical outcomes

	CFZ (*n* = 52)	CRO (*n* = 61)	*P* value
Clinical Success (%)	47 (90.4)	53 (86.9)	0.56
Hospital LOS, days (IQR)	4.0 (3.1–6.3)	3.8 (2.6–4.5)	0.05
CDI (%)	0 (0)	4 (6.6)	0.12

**Table 4:** Multivariate regression analysis for the primary outcome

Covariate	OR	95% CI	*P* value
Receipt of another antibiotic	0.46	0.11–1.83	0.27
Bacteremia	1.73	0.30–8.79	0.52
Nephrolithiasis	0.35	0.09–1.54	0.16
Urinary catheter	0.24	0.06–0.89	**0.03**
Subjective fever	0.75	0.18–2.93	0.68
Cefazolin treatment	0.46	0.10–1.86	0.28

Patients treated with cefazolin had a numerically longer median length of hospital stay than those treated with ceftriaxone (4.0 [3.1–6.3] vs. 3.8 [2.6–4.5], *P* = 0.05). No patients in the cefazolin arm, and 4/61 (7%) patients in the ceftriaxone arm, developed CDI within 30 days (*P* = 0.12) (Table 3).

Additionally, we conducted a sensitivity analysis where we excluded those cefazolin patients who received one dose of ceftriaxone prior to cefazolin therapy to eliminate this potential confounding variable. 30-day clinical success was 91% for cefazolin-treated patients versus 87% for ceftriaxone-treated patients (*P* = 0.74).

## Discussion

This is the first study to compare a narrow-spectrum IV beta-lactam antibiotic to ceftriaxone for the treatment of cUTIs in a largely male veteran population requiring hospitalization. There is little guidance currently on empiric treatment recommendations for elderly male patients that are hospitalized for cUTIs, as no IDSA guidelines exist addressing this patient population. Our study found no difference in clinical success rates between cefazolin and ceftriaxone. These findings support the use of empiric cefazolin for inpatients requiring antibiotics for cUTI in institutions with high susceptibility rates to first-generation cephalosporins. Additionally, our study is consistent with findings from other publications and, by including an older male population with urological abnormalities, expands on the scope of prior work by suggesting cefazolin as a potentially viable treatment option for cUTI treatment [4–6]. 

Outcomes were favorable even in the presence of bacteremia or other complicated clinical situations, such as nephrolithiasis. Bacteremia did not independently correlate with worse outcomes, while urinary catheters did. Patients receiving cefazolin had a longer length of hospital stay, which was not statistically significant. It is important to note that total duration of antibiotics in our study was a median of 8 days, which correlates well with recent data demonstrating that 7 days of therapy is safe and effective for patients with cUTI [8, 9]. Studies have identified preceding third-generation cephalosporin use as a risk factor for the subsequent development of CDI [10, 11]. Our study suggests a trend towards an increased incidence rate of CDI in the ceftriaxone group, compared to the cefazolin group, which had zero patients develop CDI. Our study was not powered to detect a difference in this secondary outcome.

There are limitations to consider when interpreting the results of this study. First, the retrospective nature of this observational study limits our ability to capture the many patient factors that impact treatment choice. Second, there were multiple differences in patient characteristics noted between both groups, suggesting selection bias and a preference by clinicians to choose one antibiotic over another for certain situations. Notably, the cefazolin group had significantly more non-specific symptoms, such as altered mental status, which could indicate another source of infection or worsening of co-morbidities rather than the emergence of a UTI. A pragmatic consideration was made to include altered mental status as a UTI symptom given that many elderly veteran patients with new mental status changes are treated empirically for UTI in clinical practice, particularly if urinalysis and culture findings are consistent with UTI. As such, we excluded those patients with only mental status changes reported plus negative culture findings to minimize the inclusion of patients without true infection. Third, inclusion of patients with normal flora, polymicrobial, and negative urine cultures made up 39% of the entire cohort. It is noted that some of these patients had index urine cultures that were normal flora, polymicrobial, or negative due to such circumstances as a prior dose of antibiotics, for example. Additionally, we based our selection of patients on ICD-10 codes followed by confirmation of cUTI with documented symptoms suggestive of UTI. Fourth, inclusion of patients who received up to one dose of an alternative antimicrobial, could confound the results. Significantly more patients in the cefazolin group received one dose of another antibiotic, including 37% who received one dose of ceftriaxone prior to initiating cefazolin. We allowed a single dose of another antibiotic as most single doses are administered in the emergency department, prior to hospital admission, and ceftriaxone is commonly used to meet sepsis performance measures (SEP-1) [12]. However, to minimize this impact, we excluded patients who received more than one dose of non-study antibiotics. Additionally, we conducted a multivariate analysis and included receipt of another antibiotic; this remained a non-significant predictor of achieving the primary outcome. We also conducted a sensitivity analysis where we excluded those cefazolin patients who received one dose of ceftriaxone prior to cefazolin therapy. Once again, this had no impact on the primary outcome. Fifth, the generalizability of our findings to other populations and institutions might be limited as this study was conducted in the VA system. Also, our local institutional cefazolin susceptibilities are > 90% for common UTI pathogens, while other institutions might have a lower susceptibility profile or an inability to report correct cefazolin breakpoints, such as with older Vitek^®^ panels. Lastly, this study was slightly underpowered to identify a difference between treatment arms where one truly does exist.

## Conclusions

Future research is needed to further establish the safety and efficacy of cefazolin for cUTIs. While observational studies can corroborate our findings, randomized controlled trials would provide clinicians the greatest confidence in using cefazolin for cUTIs. In conclusion, we found that patients receiving cefazolin for cUTIs experienced similar 30-day clinical success compared with those receiving ceftriaxone. Cefazolin appears worthy of broader consideration as cUTI therapy when local susceptibility data are favorable.

## Electronic supplementary material

Below is the link to the electronic supplementary material.

Supplementary Material 1

Supplementary Material 2

## Data Availability

Data is provided within the manuscript or supplementary information files. The datasets used and/or analyzed during the current study are available from the corresponding author on reasonable request.

## Abbreviations

CDI: *Clostridioides difficile* infection
CFZ: Cefazolin
CRO: Ceftriaxone
cUTI: Complicated urinary tract infection
IDSA: Infectious Diseases Society of America
IQR: Interquartile range
IV: Intravenous
UTI: Urinary tract infection
