# Supplementary material for: A comparison of empiric therapy with cefazolin versus ceftriaxone for patients with complicated urinary tract infections in a tertiary care veterans affairs medical center
Source: BMC Infect Dis. 2025 Mar 3;25:302. doi: 10.1186/s12879-025-10494-5 (PMC11874385; doi:10.1186/s12879-025-10494-5)
Supplement: Supplementary file 1 — Supplementary Material 1 [file 12879_2025_10494_MOESM1_ESM.docx]

**Supplementary Table 1.** Local urinary pathogen antibiogram comparing cefazolin* and ceftriaxone for non-ESBL isolates

|  | 2020 | | 2021 | | 2022 | |
| --- | --- | --- | --- | --- | --- | --- |
|  | CFZ | CRO | CFZ | CRO | CFZ | CRO |
| *E. coli* | 95% | 94% | 96% | 98% | 95% | 98% |
| *K. pneumoniae* | 97% | 96% | 97% | 98% | 94% | 94% |
| *P. mirabilis* | 98% | 99% | 97% | 100% | 95% | 100% |

Values represent percent susceptible.

*MIC breakpoint of ≤16 was utilized when reporting cefazolin susceptibilities for *E. coli*, *K. pneumoniae*, and *P. mirabilis* when isolated in the urine per the CLSI. *Source:* Clinical and Laboratory Standards Institute (CLSI). Performance Standards for Antimicrobial Susceptibility Testing. 32nd ed. CLSI supplement. Clinical and Laboratory Standards Institute, USA, 2022.

**Supplementary Table 2.** Discharge antibiotic regimens

|  | CFZ (n=52) | CRO (n=61) |
| --- | --- | --- |
| Beta-lactam^a^ (%) | 36 (69.2) | 40 (65.6) |
| Fluoroquinolone (%) | 5 (9.6) | 10 (16.4) |
| Sulfamethoxazole/trimethoprim (%) | 3 (5.8) | 5 (8.2) |
| Doxycycline (%) | 2 (3.8) | 0 (0) |
| No antibiotics (%) | 6 (11.5) | 6 (9.8) |

^a^Beta-lactam agents include cephalexin (n = 27 for cefazolin, n = 8 for ceftriaxone); cefpodoxime (n = 2 for cefazolin, n = 24 for ceftriaxone); cefadroxil (n = 2 for cefazolin); amoxicillin/clavulanate (n = 4 for cefazolin, n = 4 for ceftriaxone); amoxicillin (n = 1 for cefazolin, n = 4 for ceftriaxone).
