# Supplementary figures and images for: A comparison of empiric therapy with cefazolin versus ceftriaxone for patients with complicated urinary tract infections in a tertiary care veterans affairs medical center
Source: BMC Infect Dis. 2025 Mar 3;25:302. doi: 10.1186/s12879-025-10494-5 (PMC11874385; doi:10.1186/s12879-025-10494-5)

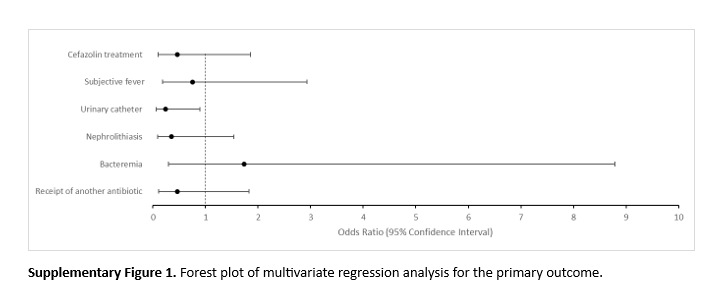

Supplement: Supplementary file 2 — Supplementary Material 2 [file 12879_2025_10494_MOESM2_ESM.png]
